# Comparison of machine learning algorithms to predict intentional and unintentional poisoning risk factors

**DOI:** 10.1016/j.heliyon.2023.e17337

**Published:** 2023-06-24

**Authors:** Yousef Veisani, Hojjat Sayyadi, Ali Sahebi, Ghobad Moradi, Fathola Mohamadian, Ali Delpisheh

**Affiliations:** a Psychosocial Injuries Research Center, Ilam University of Medical Sciences, Ilam, Iran; b Non-Communicable Diseases Research Center, Ilam University of Medical Sciences, Ilam, Iran; c Department of Epidemiology and Biostatistics, School of Medicine, Social Determinants of Health Research Center, Research Institute for Health Development, Kurdistan University of Medical Sciences, Iran; d Department of Psychology, Psychosocial Injuries Research Center, Ilam University of Medical Sciences, Ilam, Iran; e Department of Epidemiology, Faculty of Health, Safety Promotion and Injury Prevention Research Centre Shahid Beheshti University of Medical Sciences Tehran, Iran

**Keywords:** Machine learning, Intentional poisoning, Unintentional poisoning, GBT

## Abstract

**Introduction:**

A major share of poisoning cases are perpetrated intentionally, but this varies depending on different geographical regions, age spectrums, and gender distribution. The present study was conducted to determine the most important factors affecting intentional and unintentional poisonings using machine learning algorithms.

**Materials and methods:**

The current cross-sectional study was conducted on 658 people hospitalized due to poisoning. The enrollment and follow-up of patients were carried out during 2020–2021. The data obtained from patients’ files and during follow-up were recorded by a physician and entered into SPSS software by the registration expert. Different machine learning algorithms were used to analyze the data. Fit models of the training data were assessed by determining accuracy, sensitivity, specificity, F-measure, and the area under the rock curve (AUC). Finally, after analyzing the models, the data of the Gradient boosted trees (GBT) model were finalized.

**Results:**

The GBT model rendered the highest accuracy (91.5 ± 3.4) among other models tested. Also, the GBT model had significantly higher sensitivity (94.7 ± 1.7) and specificity (93.2 ± 4.1) compared to other models (P < 0.001). The most prominent predictors based on the GBT model were the route of poison entry (weight = 0.583), place of residence (weight = 0.137), history of psychiatric diseases (weight = 0.087), and age (weight = 0.085).

**Conclusion:**

The present study suggests the GBT model as a reliable predictor model for identifying the factors affecting intentional and unintentional poisoning. According to our results, the determinants of intentional poisoning included the route of poison entry into the body, place of residence, and the heart rate. The most important predictors of unintentional poisoning were age, exposure to benzodiazepine, creatinine levels, and occupation.

## 1 Introduction

Exposure to toxic substances, especially chemicals, is a serious and urgent problem worldwide. Global industrialization, as well as the expansion of various types of chemicals are among the factors exaggerating concerns in this regard [1].

The Global Burden of Disease Study (2019) shows that unintentional poisonings alone are responsible for 8755 annual deaths and the loss of 696,767 disability-adjusted life years. This report revealed a decline in unintentional poisoning cases from 1990 to 2019, which was parallel with 30.3% and 28.8% reductions in the total mortality rate and disability-adjusted lost life years, respectively, in 2019 compared to 1990 [2].

In Iran, following global trends, there has been a decline in the rate of unintentional injuries, reaching 62 deaths per 100,000 population, showing a decrease of about 77% compared to 1990 [3]. On the other hand, according to studies over a decade, the rate of acute poisoning in men has increased by 70%, showing a 10-fold increase [4]. Intentional poisoning is more commonly reported in women, 22% of whom reporting a history of suicide attempts, with the poisoning drug belonging to neuropsychiatric drugs in 50% of cases [5]. In unintentional poisoning cases, cardiovascular drugs comprise the most important agents [6].

A noteworthy portion of poisoning cases are perpetrated intentionally, varying depending on different geographical regions and the age and gender distribution of the population. Poisons such as organophosphorus, carbamate, organochlorine, paraquat, and aluminum phosphide are the main poisons used intentionally in rural areas, which usually cause a high mortality rate. In urban areas, mediations are more commonly used, which often are associated with a lower mortality. According to the statistics, deaths caused by intentional poisoning reach 0.5–1% in developed countries and 10–20% in developing countries [7].

In a study conducted on adults showed that intentional poisoning was more likely to inflict harm. As well, those perpetrating intentional poisoning were more likely to be jobless and have a lower IQ [8]. Also, in another study, among the most important risk factors for unintentional poisoning were reported to have drug addicted members in the family and a history of previous attempts of poisoning [9].

The aim of the present study was to determine the most important factors influencing intentional and unintentional poisoning using machine learning algorithms, therefore we assessed goodness of fit of different machine learning models to obtain of reliable predictor model for identifying the factors affecting intentional and unintentional poisoning.

## 2 Materials and methods

This was a cross-sectional-analytical study conducted in Ilam, the capital city of Ilam province, and in a university-affiliated hospital. Ilam province harbors a population of over 630 thousand people, and Ilam city has a population of 240 thousand people. Currently, all patients presenting with poisoning symptoms are referred to the only university-affiliated hospital in Ilam city. Patients are initially examined in the emergency unit, and after undergoing additional testing, if poisoning is diagnosed, they are admitted to the hospital's poisoning ward.

Our participants included all the patients diagnosed with poisoning and admitted to the poisoning ward of the hospital. The registration of patients and entering information in the statistical software lasted two consecutive years (2020 and 2021). The data were gathered by reviewing patient files and follow-ups by a doctor and entered into the statistical software by an expert. Inclusion criteria for this study were the clinical diagnosis of poisoning by a physician and admission to the poisoning ward of the hospital. Exclusion criteria encompassed incomplete patient files, the absence of serological tests’ results, and lack of approval by a clinician.

The data gathered were demographic information, clinical findings, and the results of serological tests. All cases of poisoning should have been approved by a clinician. In this study, the data gathered included age, gender, level of education, dates of registration/hospitalization/discharge, location of poisoning, place of residence, vacancy, job, marital status, income, route of poisoning, consciousness level, treatments administered, respiration and heart rate, systolic/diastolic blood pressure, body temperature, the type of the poisoning agent (tricyclic antidepressants (TCI), aluminum phosphide (ALP), methadone (MTD), morphine, acetaminophen, benzodiazepine (BZO), non-steroidal anti-inflammatory drugs (NSAID), methamphetamine, amphetamine, poisons used in agriculture and animal husbandries, animal poisons, alcoholic drinks, tramadol (TML), steroid drugs, antipsychotics, antibiotics, and antimanic drugs), drug dosage, history of drug abuse, history of diseases and psychological problems, history of addiction and the type of the narcotic, and history of attempted suicide.

### 2.1 Data cleansing

The preprocessing of the data, including data cleaning and formatting, is a vital and very challenging step during data mining. Here, we excluded from the list of analytic variables those that had a Pearson correlation coefficient above 90%. In addition, variables with 50% missing values and stability above 90% were removed from the list of variables. Also, the presence of duplicate data and unusual values was checked. The data remaining were considered as the processed data set for this study.

### 2.2 Model evaluation

Since the response variable was binary (0 for unintentional poisoning and 1 for intentional poisoning), the algorithms used in this study were logistic regression (LR), deep learning (DL), gradient boosted trees (GBT), and support vector machine (SVM). For all data sets, 10-fold cross-validation was used. During each fold, the models presented were fitted on the learning data, and then the observations were used for validation. After fitting the models on the training data, the models were evaluated using the criteria of accuracy, sensitivity, specificity, F measure, and the area under the rock curve (AUC). In the following, we briefly explained the algorithms used in this research.

Gradient Boosted Trees (GBT) is a machine learning algorithm that is used for both regression and classification problems. It is an ensemble method that combines multiple decision trees to make predictions. The algorithm works by iteratively adding decision trees to the model, with each new tree attempting to correct the errors made by the previous trees.

The GBT algorithm can be broken down into the following steps:

1. Initialize the model with a single decision tree.
2. Make predictions on the training data using the current model.
3. Calculate the errors between the predicted values and the actual values.
4. Fit a new decision tree to the errors, with the goal of minimizing the errors.
5. Add the new decision tree to the model.
6. Repeat steps 2–5 until the desired number of trees have been added to the model.

The GBT algorithm uses a gradient descent optimization technique to minimize the errors. The gradient descent algorithm works by iteratively adjusting the model parameters in the direction of the negative gradient of the loss function. The loss function is a measure of how well the model is performing, and the negative gradient indicates the direction in which the loss function is decreasing.

This algorithm uses a specific loss function called the deviance, which is a measure of the difference between the predicted values and the actual values. The deviance is calculated as the negative log-likelihood of the Bernoulli or multinomial distribution, depending on whether the problem is a binary or multi-class classification problem. It also includes a regularization parameter, which helps to prevent overfitting. The regularization parameter controls the complexity of the model by penalizing large values of the model parameters. This helps to ensure that the model is not too complex and is able to generalize well to new data.

### 2.3 Gradient boosted trees

The GBT algorithm is a special type of random forest algorithm, in which instead of producing parallel decision trees, these trees are created serially and sequentially, improving the performance of the tree at each step, meaning that each tree at each step is consecutively produced based on the mistakes of the previous tree. This process reduces the prediction errors of the model, leading to the creation of a potent learner. GBT compared to other random forest algorithm creates multiple decision trees, but each tree is trained sequentially. The algorithm starts with a weak learner and then adds more trees to improve the model's performance. Each subsequent tree is trained to correct the errors made by the previous tree.

### 2.4 Data analysis

Quantitative data were summarized using mean, standard deviation, median, maximum, and minimum, and qualitative data were described by frequency and percentage. Data analysis was conducted in Stata 11 (StataCorp, LLC College Station, Texas, USA) and RapidMiner 9.10 (RapidMiner Studio) software. RapidMiner Studio is a data platform developed based on JAVA programming and is used for educational analysis and research purposes in data mining and artificial intelligence. The program is equipped with statistical tools and a machine learning toolbox composing several AI predictive algorithms used for supervised and unsupervised learning purposes. In this research, a version operating under Windows 10 of this software with an Intel processor (Intel (R) Core (TM) i7-5500U CPU @ 2.40 GHz, 2 cores), and four logical processors was used.

## 3 Results

The medical profiles of 658 individuals admitted due to poisoning were assessed, 460 (69.5%) and 198 (30.1%) of whom were intentional and unintentional cases, respectively. The mean age was 26.1 ± 6.9 years in those with intentional poisoning and 34.7 ± 7.5 years in people with unintentional poisoning.

According to the data obtained from machine learning models, the average of precision in all four models was 80.6%, and the GBT model showed the highest precision (91.5 ± 3.4%) among the models tested. In terms of sensitivity, all four models had acceptable performance, and in terms of specificity, the GBT model had better functionality (0.73) compared to other models. In the final analysis, the GBT model acquired a higher average in terms of sensitivity and specificity than other models, so it was adopted as the model of choice (Table 1).

**Table 1:** Performance Metrics for four machine learning models.

Models	Accuracy	AUC^a^	F Measures	Sensitivity	Specificity
Logistic Regression	79.8	0.954	87.3	100	31.8
Deep Learning	80.9	0.913	87.6	99.3	41.5
Gradient Boosted Trees	91.5	0.967	93.9	94.7	83.2
Support Vector Machine	70.2	0.513	82.4	100	28.3

a Area under the ROC (receiver operating characteristic) curve.

**Fig. 1:**
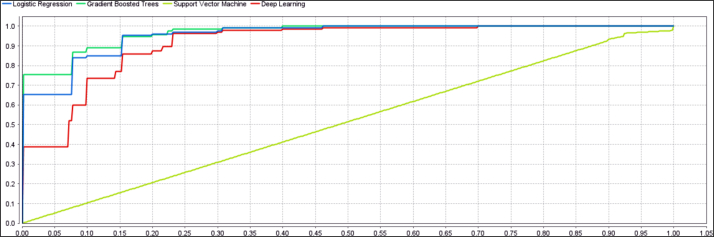
ROC cure for the proposed model by cross validation.

For all four models, ROC curve analysis was performed to assess the ability (or sensitivity) of each model to identify people perpetrating intentional poisoning. Based on proximity to the upper corner of the curve (or the area under the curve), the ability of models in identifying people with poisoning can be compared. According to this, the AUC (2.1 ± 0.967) of the GBT model was higher compared to those of other models, indicating that this model was more capable of detecting individuals with intentional poisoning, reflecting its superior performance (Fig. 1).

**Graph 1:**
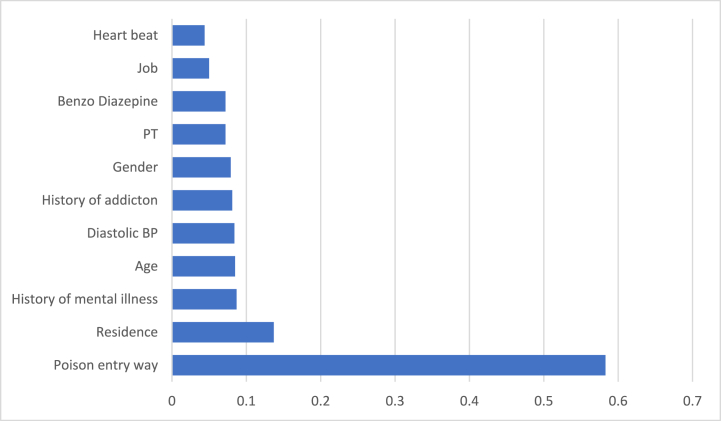
The wight of main covariate by GBT model.

According to the results of the GBT model, the most important predictors of unintentional poisoning were the route of poison entry (weight = 0.583), place of residence (weight = 0.137), history of psychiatric disorders (weight = 0.087), and age (weight = 0.085). Other influential variables, in order of their impact, included diastolic blood pressure, history of addiction, gender, use of benzodiazepine, job, and the heart rate (Graph 1).

**Graph 2:**
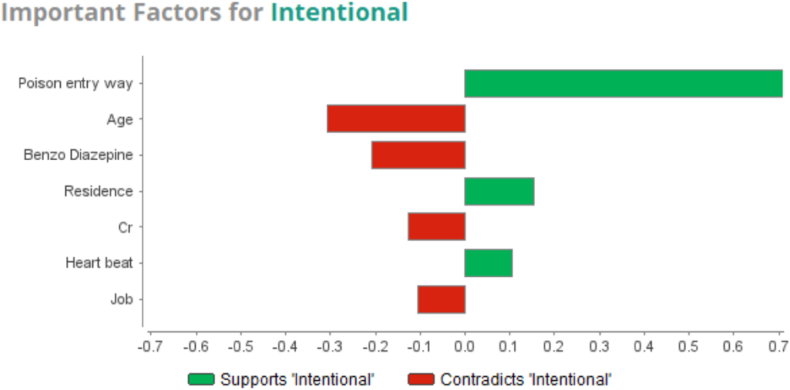
Important factors for intentional and unintentional by GBT model.

According to the GBT model, the most important predictors of intentional poisoning encompassed the route poison entry, place of residence, and the heart rate, as well as the factors previously known to predict unintentional poisonings, including age, use of benzodiazepines, creatinine level, and occupation (Graph 2).

**Graph 3:**
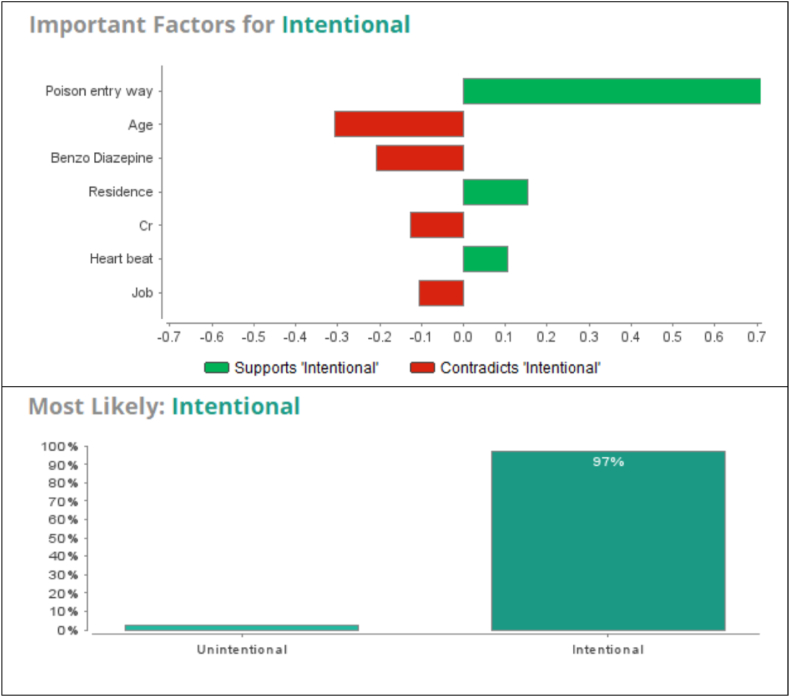
important factors for intentional and unintentional by GBT model in.

Analysis based on the GBT model was performed using hypothetical input variables related to a 17-year-old single high school student girl with no history of psychological problems, a family with an average economic level, living in a village, whose route of poison entry was orally. Based on the results, the GBT model predicted intentional poisoning for this individual by 97% probability. According to this hypothetical model, the variables that had the greatest impact on the prediction of intentional poisoning were the route of poison entry, place of residence, and heart rate. Also, the most important predictors of unintentional poisoning included occupation, creatinine levels, use of benzodiazepine, and age (Graph 3).

## 4 Discussion

The present study was conducted on the basis of registration data, in which we prospectively evaluated the patients hospitalized due to poisoning. The aim of this study was to ascertain the factors influencing intentional and unintentional poisoning in these individuals. The results of this study showed that the frequency of intentional poisoning (69.5%) among the hospitalized patients was twice the rate of unintentional poisoning. Also, people perpetrating intentional poisoning were on average eight years younger than those affected by unintentional poisoning, showing a higher rate of intentional poisoning in young adults. A study based on the data of the National Poison Data System (NPDS) in the United States revealed that only 26% of cases of acetaminophen poisoning were referred to medical centers and the fact that liver damage in these people was more pronounced compared to the public [10]. A study in Jordan showed that among adult people referring to medical centers due to poisonings, 25.3% were intentional; 93% of the cases occurred either at home or at workplace, and 94% of the people were poisoned via the oral route [11]. In another study in South Korea, 59.9% of poisoning cases were intentional, 73% and 27% of which were reported in people younger and older than 65 years old, respectively [12]. The frequency of intentional poisoning among hospitalized patients in the United States was reported to be 43.3% [8].

The results of the GBT model showed that the variables that had the highest weight in determining the predicting power of the model included the route of poison entry, place of residence, history of psychiatric conditions, and the patient's age. Other influential variables in the model were high diastolic blood pressure, history of addiction, gender, benzodiazepine, occupation, and heart rate. Mehrpour et al., in their study on 2878 cases of metformin poisoning, investigated the determinants of the incidence of poisoning using a decision tree model and reported that acidosis, hypoglycemia, and hypotension were the most prominent predictors in this model [16]. In another study, Oh et al. investigated 4023 individuals, where the most important predictors of intentional poisoning according to a decision tree model were found to be alcohol consumption (34.1%), cigarette smoking (16.5%), physical illnesses (13.0%), advanced age (11.9%) and suffering from psychiatric disorders (7.3%) [12].

Based on our literature search, this was the first report using machine learning models to recognize the predictors of intentional and unintentional poisoning in patients. The results of model analysis showed that the GBT model could reliably identify the predictors of intentional and unintentional poisoning in patients. Data mining techniques can help researchers and clinicians design interventions and make effective clinical decisions. The GBT model offers one of the most important data mining models in which, instead of creating parallel decision-making trees, these trees are formed serially and sequentially, where the performance of the tree is upgraded at each step. In other words, each consecutive tree at each step is generated based on the errors of the prior tree, which reduces the model's prediction errors and creates a more robust learner. In a study, Amirabadizadeh et al. used machine learning models to identify people at risk of arbitrarily use of medications, and reported that logistic regression analysis delivered more accurate results [13]. In another study, Potash et al. employed machine learning models to predict poisoning in children [14]. In other studies, machine learning models have also offered high accuracy, sensitivity, and specificity for reliably determining the risk of poisoning [12,15].

According to our findings in the present study, the most important predictors of intentional poisoning in our patients were the route of poison entry into the body, place of residence, and heart rate. Rancic et al. declared that 95% of intentional poisoning cases were due to medication abuse, and 95% of the individuals hospitalized due to intentional poisoning had no history of alcohol consumption [8]. Farzaneh et al. also investigated the risk factors of intentional poisoning with aluminum phosphide and declared that high systolic blood pressure and bicarbonate level were the most important risk factors of mortality in these patients [17].

According to the results of the present study, the most important predictors of unintentional poisoning were the patient's age, use of benzodiazepine, creatinine level, and occupation. In accordance, some studies have noted that people with unintentional poisoning had elevated levels of creatine [8]. In addition, Figgatt et al. noted benzodiazepine consumption as responsible for 9% of the deaths caused by unintentional poisoning, where 53.7% of the victims had an age between 15 and 34 years old [18].

Among the strengths of the study, it can be mentioned that the present study was conducted based on patient registrations, so the data source is valid. All patients have been visited by a clinical specialist and the toxicology results have been confirmed by a pharmacology specialist. Also, machine learning models were used to investigate the determinants of the poisoning instead of models in classical statistics.

## 5 Conclusion

The present study showed that GBT was a valid predictive model to investigate the factors affecting intentional and unintentional poisoning in hospitalized patients. The most important predictors of intentional and unintentional poisoning according to the GBT model were the route of poison entry, place of residence, history of psychiatric conditions, and the patient's age. Our results affirmed the findings of previous studies regarding the importance of predictors of intentional poisoning in patients. Special attention should be paid to the route of poisoning, history of psychiatric disorders in patients, and younger ages as major risk factors of intentional poisoning.

## Declarations

### Ethics statement

This study resulted from the intentional poisoning registration program approved by the Deputy of Research and Technology of Ilam University of Medical Sciences and received the ethics code of IR.MEDILAM.REC.1398.055.

### Author contribution statement

Yousef Veisani: Conceived and designed the experiments; Performed the experiments; Analyzed and interpreted the data and wrote the paper.

Hojjat Sayyadi: Analyzed and interpreted the data; Performed the experiments; and wrote the paper.

Ali Sahebi; Fathola Mohamadian: Contributed reagents, materials, analysis tools or data and wrote the paper.

Ghobad Moradi: Conceived and designed the experiments; and wrote the paper.

Ali Delpisheh: Conceived and designed the experiments; Contributed reagents, materials, analysis tools or data; and wrote the paper.

### Data availability statement

Data will be made available on request.

## Declaration of competing interest

The authors declare that they have no known competing financial interests or personal relationships that could have appeared to influence the work reported in this paper.

## References

[1] Binetti R., Costamagna F.M., Marcello I. (2008). Exponential growth of new chemicals and evolution of information relevant to risk control. Ann. Ist. Super Sanita.

[2] Peden A.E., Cullen P., Francis K.L., Moeller H., Peden M.M., Ye P. (2022). Adolescent transport and unintentional injuries: a systematic analysis using the Global Burden of Disease Study 2019. Lancet Public Health.

[3] Ou Z., Yu D., Liang Y., Wu J., He H., Li Y., He W., Gao Y., Wu F., Chen Q. (2022 Jun 11). Global burden of rheumatic heart disease: trends from 1990 to 2019. Arthritis Res. Ther..

[4] Asadi R., Afshari R. (2016). Ten-year disease burden of acute poisonings in northeast Iran and estimations for national rates. Hum. Exp. Toxicol..

[5] Masoumi G., Ganjei Z., Teymoori E., Sabzghabaee A.M., Yaraghi A., Akabri M. (2013). Evaluating the prevalence of intentional and unintentional poisoning in vulnerable patients admitted to a referral hospital. J. Isfah. Med. Sch..

[6] Yen C.W., Lee E.P., Cheng S.C., Hsia S.H., Huang J.L., Lee J. (2021 Nov). Household cleaning products poisoning in a pediatric emergency center: a 10- year cross-sectional study and literature review. Pediat. Neonatol..

[7] Hawton K., Ferrey A., Casey D., Wells C., Fuller A., Bankhead C. (2019). Relative toxicity of analgesics commonly used for intentional self-poisoning: a study of case fatality based on fatal and non-fatal overdoses. J. Affect. Disord..

[8] Rancic N., Rankovic A., Savic D., Abramovic A., Rancic J., Jakovljevic M. (2015). Intentional self-poisonings and unintentional poisonings of adolescents with nonfatal outcomes. J. Child Adolesc. Subst. Abuse.

[9] Mansori K., Soori H., Farnaghi F., Khodakarim S., Mansouri Hanis S., Khodadost M. (2016). A case-control study on risk factors for unintentional childhood poisoning in Tehran. Med. J. Islam. Repub. Iran.

[10] Brass E.P., Burnham R.I., Reynolds K.M. (2019). Poison center exposures due to therapeutic misuse of nonprescription acetaminophen-containing combination products in the United States 2007–2016. Clin. Toxicol..

[11] Beauchamp G.A., McKeown N.J., Rodriguez S., Spyker D.A. (2016 Mar). Relating calls to US poison centers for potential exposures to medications to Centers for Disease Control and Prevention reporting of influenza-like illness. Clin. Toxicol..

[12] Oh E.S., Choi J.H., Lee J.W., Park S.Y. (2018). Predictors of intentional intoxication using decision tree modeling analysis: a retrospective study. Clin. Exp. Emerg. Med..

[13] Amirabadizadeh A., Nakhaee S., Mehrpour O. (2022). Risk assessment of elevated blood lead concentrations in the adult population using a decision tree approach. Drug Chem. Toxicol..

[14] Potash E., Ghani R., Walsh J., Jorgensen E., Lohff C., Prachand N. (2020). Validation of a machine learning model to predict childhood lead poisoning. JAMA Netw. Open.

[15] Stoia M., Kurtanjek Z., Oancea S. (2016). Reliability of a decision-tree model in predicting occupational lead poisoning in a group of highly exposed workers. Am. J. Ind. Med..

[16] Mehrpour O., Saeedi F., Hoyte C., Goss F., Shirazi F.M. (2022). Utility of support vector machine and decision tree to identify the prognosis of metformin poisoning in the United States: analysis of National Poisoning Data System. BMC Pharmacol. Toxicol..

[17] Farzaneh E., Ghobadi H., Akbarifard M., Nakhaee S., Amirabadizadeh A., Akhavanakbari G. (2018). Prognostic factors in acute aluminium phosphide poisoning: a risk-prediction nomogram approach. Basic Clin. Pharmacol. Toxicol..

[18] Hoots B., Vivolo-Kantor A., Seth P. (2020 May). The rise in non-fatal and fatal overdoses involving stimulants with and without opioids in the United States. Addiction.

